# Baked Bread Enhances the Immune Response and the Catabolism in the Human Body in Comparison with Steamed Bread

**DOI:** 10.3390/nu12010001

**Published:** 2019-12-18

**Authors:** Huisong Wang, Guangchang Pang

**Affiliations:** 1 Tianjin Key Laboratory of Food Biotechnology, College of Biotechnology and Food Science, Tianjin University of Commerce, Tianjin 300134, China; 2 College of Animal Science and Technology, Nanjing Agricultural University, Nanjing 210095, China

**Keywords:** Baked bread, Cytokine network, Human, Metabolic network, Steamed bread

## Abstract

It is unclear whether different processing methods change the biological functions of foods and how these functions are evaluated in the human body. Here, steamed bread and baked bread, the traditional staple foods in China and many Western countries, were made by steaming and baking, respectively, using one piece of fermented wheat dough and then consumed by 16 healthy young volunteers. By detecting 38 cytokines, 12 metabolic enzymes, glucose, lactate, and nicotinamide adenine dinucleotide (NADH) in the serum, the cytokine network and central metabolic pathway network were investigated to compare the effects of the two staple foods on immunity and metabolism. Compared with steamed bread, baked bread increased (*p* < 0.05) concentrations of fractalkine and macrophage-derived chemokine, decreased (*p* < 0.05) the concentration of interleukin-1RA, increased (*p* < 0.05) the expression level of phosphofructokinase, and decreased (*p* < 0.05) the expression level of glucose-6-phosphate dehydrogenase in the serum. Two network analyses indicated that baked bread, as compared to the steamed bread, enhanced communication between immune cells, increased catabolism, and decreased anabolism. Further, a correlation analysis of cytokines and metabolic enzymes suggested that the two staple foods may affect metabolism by regulating the secretion of cytokines. These findings highlight how the same raw food material processed by different methods may have different impacts on immunity and metabolism in humans.

## 1. Introduction

Highly complex chemical changes occur as a result of different food-processing methods (e.g., steaming, baking, frying, etc.) [1], which also have different effects on the physiologic functions of the human body after they are ingested [2,3]. Researchers have investigated changes in certain components of foods after they undergo these different processing methods [4,5]. However, it is difficult to identify the changes different processing methods cause in all components, particularly those changes that occur after ingestion, and understand the interactions between the components and the gastrointestinal tract [6]. Therefore, it is more meaningful for human health to explore the effects of foods processed through different methods on the human body than to study the chemical changes that occur because of different processing techniques. Steamed bread and baked bread are the staple foods of people in China and Western countries, respectively. They are both made from fermented wheat dough. However, the former is made by steaming the dough at 100 °C, the latter is made by baking the dough at 180–230 °C. The same wheat dough processed by these two different methods may have different impacts on the immune, endocrine, and metabolic systems of the human body. However, as far as we know, no research has been conducted on this subject. Understanding the effects of these two staple foods on human physiological functions is important for exploring the effects of food-processing methods on the human body and for dietary intervention to promote the human health.

After ingestion, nutrients and non-nutrients can both affect human physiological functions. Recently, an increasing amount of research has indicated that the non-nutrients of foods play important roles in regulating immunity and metabolism via signaling pathways, roles that are mainly mediated by the intestinal mucosa [7,8,9]. Different food-processing methods cause a variety of non-nutritional chemical changes. Therefore, the influence of steamed bread and baked bread made from one piece of fermented wheat dough on human physiological functions may be more from the signaling processes of non-nutrients than from the nutrients themselves.

For the functional evaluation of foods, the majority of studies have been conducted on animals. However, no animal can truly reflect the effects of foods on the human body. Through extensive research, we have established the cytokine network and central metabolic pathway (CMP) network to quantitatively evaluate the effects of foods on immunity and metabolism in the human body [8,10,11,12,13,14]. 

In the human body, blood circulation facilitates the exchange of material, energy, and information throughout the whole body. Cytokines, produced by the specific cytokine-producing cells, bind to specific receptors on immune and nonimmune cells through the circulatory system, which forms an intercellular communication network—that is, the cytokine network [8,15,16]. This network regulates neuro-immune-endocrine functions and metabolism. Moreover, because of the complexity of cytokine signaling, it is hard to understand the actual functions of a single cytokine. Therefore, the study of cytokine networks can quantitatively simulate intercellular communication, including interimmune cell communication, i.e., the systemic immune response [15,17]. Higher animals depend on oxidative phosphorylation to provide energy. Although there is the circulatory system, certain parts of the body still have a restricted oxidative respiratory chain. To ensure the redox equilibrium, pyruvate needs to be converted to lactate, and the latter continuously circulates through the blood [18]. Circulating lactate regulates oxidative phosphorylation [19,20] and adjusts the ratio of nicotinamide adenine dinucleotide (NADH)/NAD^+^ [21]. Lactate metabolic flux (the rate in the steady state) reflects the contributions of circulating lactate to the redox state and energy supply [21], and thus it has become an important indicator of systemic catabolism and oxidative phosphorylation [13,22,23,24]. Moreover, the metabolic flux entering the pentose phosphate pathway (PPP) reflects the anabolic level to some extent, as the PPP provides the raw materials and reducing power for anabolism [25]. Therefore, by constructing a CMP network, including the Embden–Meyerhof–Parnas pathway (EMP), the tricarboxylic acid cycle (TCA), and PPP, and assessing it through metabolic flux analysis, the catabolism and anabolism of the human body can be quantified to a large extent.

In this study, the goal was to compare the effects of steamed bread and baked bread made with one piece of fermented wheat dough on immunity and metabolism in the human body by using cytokine network and metabolic network models. The results showed that baked bread enhanced systemic immune response, increased catabolism, and decreased anabolism as compared with steamed bread. These findings provide critical evidence for understanding the effects of foods processed through different methods on the physiological functions of the human body for developing future dietary intervention to promote human health.

## 2. Materials and Methods

### 2.1. Making Steamed Bread and Baked Bread

Wheat flour without any additives was purchased from Dacheng Food Co., Ltd. (Tianjin, China). Two hundred grams of wheat flour and 2 g of dry yeast (Angel Yeast Co., Ltd., Yichang, China) were mixed into 48 mL of water at 38 °C. The dough was kneaded evenly by hand and leavened in a fermentation cabinet (SM-40SP, Sinmag Equipment, Wuxi, China) at 8 °C and 85% relative humidity for 60 min. Then, the dough was kneaded again for 3 min and rested for 15 min [26]. Sixteen pieces of dough were made using the same method, and each was equally divided into two. One was steamed in a steamer basket at 100 °C for 20 min to make the steamed bread according to Zhang and Wang [26]; the other was baked in an electric oven (SM-606T, Sinmag Equipment, Wuxi, China) at 215 °C for 20 min to make the baked bread according to Wronkowska et al. [27].

### 2.2. Volunteers

Sixteen healthy college students (23–25 years old, 8 men and 8 women who were not menstruating; the body mass index of male and female students were in the ranges 20.75–21.36 and 19.31–19.93, respectively) were recruited for the study. These volunteers mainly ate rice as their daily staple food and rarely ate wheat flour-based food. Volunteers were required to have regular hours of rest and study without any strenuous exercise, alcohol, caffeine, functional food, or medicine during the study period [10,11]. They all stayed in a relaxed environment to protect them from stress sources [28]. The study followed the Declaration of Helsinki. All the volunteers signed informed consent forms and liability agreement. The local ethics committee (Ethics Committee of Tianjin University of Commerce) approved this study.

### 2.3. Treatments

The experiment lasted for 5 days (Friday + first weekend + second weekend). On the first day (Friday: the transitional period), volunteers were provided with the unbiased food (Boiled potato, celery cabbage, and edible rape without oil and salt; purified water) + rice (staple food) for three meals (the amount of food was freely available). The time for three meals: breakfast was available at 07:30, lunch was at 12:00, and dinner was at 17:30. Each meal lasted 25–35 min. On the second day (first Saturday), volunteers were also provided with unbiased food + rice for three meals, and blood was collected as control. Venous blood was collected from each subject at 14:30, 15:00, and 15:30 by trained technicians; with 5 mL of blood being taken at each time point. After incubation of 2 h at 4 °C, the blood sample was centrifuged at 3000 × *g* for 10 min at 4 °C, and stored at −20 °C for future analysis. On the third day (first Sunday), breakfast was the same as on the first two days. Then, the unbiased food + 100 g steamed bread (replacing the rice) were provided for lunch; and a blood sample was collected (time and method as above). Then, a “wash out” period (a 5 day interval) was taken. During the interval, volunteers resumed their daily eating habits. On the fourth day (second Saturday), the unbiased food + rice was provided for the three meals just like the first day (transitional period). On the fifth day (second Sunday), unbiased food + 100 g baked bread were provided for lunch; and a blood sample was collected as above. The selection of blood collection time points was based on the assumption that the foods/functional foods have a relatively significant effect on the human body (including cytokines and metabolic enzymes) 2.5–3.5 h after eating [10,11,12,13,29].

### 2.4. Cytokine Network

#### 2.4.1. Detection of Serum Cytokine Profiles

Magnetic bead-based human cytokine profiling kits (HCYTMAG-60K-PX38, Millipore Corporation, MA, USA) and Luminex 200 (Millipore Corporation, MA, USA) were used to measure the concentrations of 38 cytokines in the serum obtained at 15:00 each day according to manufacturer’s instructions [30]. This time point corresponds to the time point measured for enzyme expression. The cytokines measured were granulocyte colony-stimulating factor (G-CSF), granulocyte macrophage-colony-stimulating factor (GM-CSF), fractalkine, interferon-γ (IFN-γ), IFN-α2, growth-related oncogene (GRO), macrophage-derived chemokine (MDC), monocyte chemotactic protein-1 (MCP-1), MCP-3, interferon-inducible protein-10 (IP-10), macrophage inflammatory protein-1α (MIP-1α), MIP-1β, tumor necrosis factor-α (TNF-α), TNF-β, interleukin-1α (IL-1α), IL-1β, IL-1RA, IL-2, IL-3, IL-4, IL-5, IL-6, IL-7, IL-8, IL-9, IL-10, IL-12P40, IL-12P70, IL-13, IL-15, IL-17A, vascular endothelial cell growth factor (VEGF), epidermal growth factor (EGF), fit-3 ligand, soluble CD40 ligand, fibroblast growth factor 2, eotaxin, and transforming growth factor-α; and the minimum detectable concentrations (pg/mL) of them were 2.87, 5.81, 24.02, 2.65, 2.48, 4.46, 13.60, 4.38, 8.63, 9.77, 4.13, 1.71, 1.69, 1.26, 7.86, 0.82, 5.27, 2.45, 1.30, 2.88, 2.19, 3.06, 2.81, 1.50, 0.78, 2.37, 3.86, 3.73, 1.99, 1.18, 2.07, 126.10, 9.46, 0.64, 7.25, 11.74, 0.92, and 0.40, respectively.

#### 2.4.2. Construction of Cytokine Network

The cytokine network was constructed according to previous studies [10,13,31,32]. In brief, the secretory and target cells of significantly changed (*p* < 0.05) cytokines after the intake of steamed bread and baked bread (versus control) were obtained from a cytokine database (COPE, http://www.copewithcytokines.de/) [33]. Arranged alphabetically, this database defines the function of each known cytokine, covering topics ranging from immunity to hematology, apoptosis, bioassays, and others [33]. The secretory cells and target cells of a given cytokine can be found according to its defined functions. The communication (direction and strength) among secretory cells and target cells can be calculated through Formula (1):

(1) $$E_{st}^{}/\%=\sum_{i=1}^{m=38}p_{i}s_{i}t_{i}\frac{1}{16}\sum_{j=1}^{n=16}\frac{cij-cij0}{cij0}\times 100,$$

where variable *c_ij_* denotes the concentration of cytokine *i* (one of 38 cytokines) after the intake of steamed bread or baked bread, *c_ij0_* denotes the concentration of cytokine *i* in the control, and 16 denotes the 16 sample repeats (16 volunteers). Therefore, $\frac{1}{16}\sum_{j=1}^{n=16}\frac{cij-cij0}{cij0}$ represents the average concentration change rate of cytokine *i*. The variable *p* denotes the statistical significance of cytokine *i* (versus control), with a value of 1 representing statistical significance (*p* < 0.05) and 0 representing statistical insignificance; *s* denotes whether a cell was the secretory cell of cytokine *i*, with a value of 1 for yes or 0 for no; and *t* denotes whether a cell was the target cell of cytokine *i*, with a value of 1 representing stimulation, -1 representing inhibition, or 0 representing that the cell was not a cytokine target. Then, the cytokine network diagram was drawn using Visio 2013 (Microsoft) according to the calculated *E_st_*.

This network is a directed-weighted network [34]. Secretory and target cells are nodes, the signal communications (*E_st_*) with direction and strength (thickness of line) are lines. The strength of each node was the sum of the strengths of the lines connected to the node; each node’s input strength represents the degree to which this cell is activated or inhibited by other cells. Total network strength (*St*) was the sum of the strengths of all lines, which is an important index to reflect the intercellular communication or systemic immune response [13,15]. This virtual network model simplifies complex biological processes.

### 2.5. Central Metabolic Pathway (CMP) Metabolic Network

#### 2.5.1. Construction of Metabolic Network

**Figure 1:**
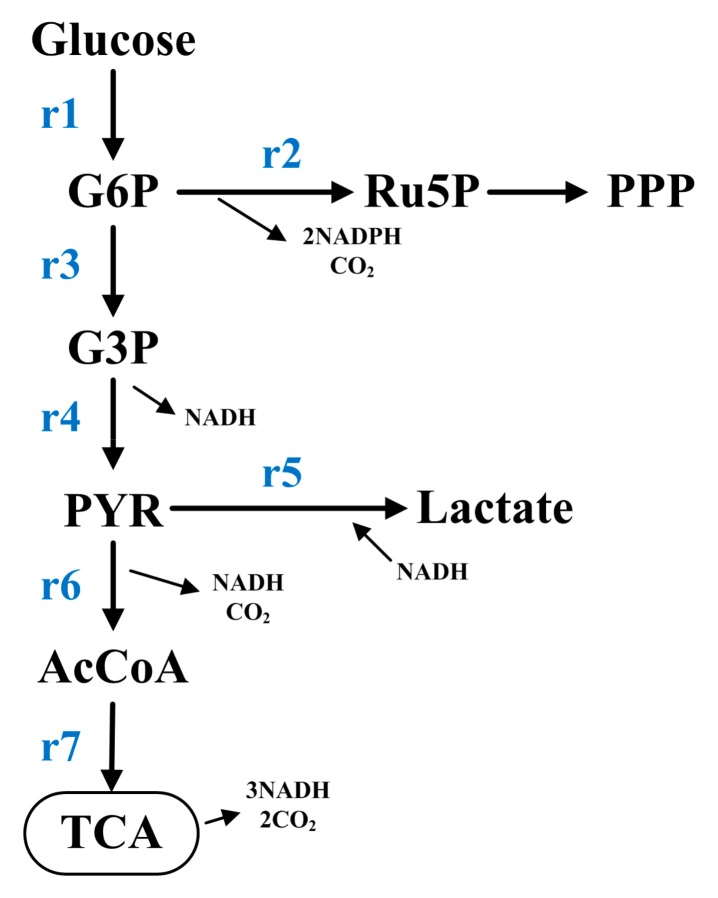
Central metabolic pathway metabolic network diagram containing the Embden–Meyerhof–Parnas (EMP) pathway, phosphopentose pathway (PPP), and tricaboxylic acid (TCA) cycle pathway. Ac-CoA, acetyl coenzyme A; G3P, glyceraldehyde-3-phosphate; G6P, glucose-6-phosphate; PYR, pyruvate; Ru5P, ribulose-5-phosphate.

The CMP metabolic network, including the EMP, PPP, and TCA (Figure 1), was constructed according to the metabolic network construction principle [35]. In this network, seven metabolic pathway fluxes (r1−r7) need to be obtained. According to the calculation principles of metabolic flux [35], four intermediate metabolites (Table 1) can be considered to have no accumulation because they can be assumed to all be in the pseudo-steady state; so their corresponding mass balance equations (Table 1) are equal to zero. Then, according to the calculation method of the matrix, the entire network, including seven metabolic pathways, can be obtained if the other three (7 − 4 = 3) metabolites in this network were detected. The metabolic rates of glucose (r1), lactate (r5), and NADH (*p* = r4 − r5 + r6 + 3r7) can be determined. Therefore, the flux in each metabolic pathway can be calculated through Formula (2). The final metabolic flux values were normalized relative to r1 (defined as 100). Visio 2013 (Microsoft Corporation, WDC, USA) was used to draw the metabolic network diagram. This network is also a directed-weighted network. The thickness of the lines represents the metabolic flux value; the direction of arrows represents the metabolic flux direction [13]. The main role of metabolic flux is the quantitative comparisons of metabolic state between the groups [36].

(2) $$\left[\begin{array}{c} r2\\ r3\\ r4\\ r6\\ r7 \end{array}\right]=\left[\begin{array}{ccc} 1 & -0.5 & -0.1\\ 0 & 0.5 & 0.1\\ 0 & 1 & 0.2\\ 0 & 0 & 0.2\\ 0 & 0 & 0.2 \end{array}\right]\times \left[\begin{array}{c} r1\\ r5\\ P \end{array}\right]$$

#### 2.5.2. Determination of Serum Glucose and Lactate

The serum samples collected at 14:30 and 15:30 were used to detect the content of glucose and lactate by using a SBA−40C biosensor analysis meter (Biotechnology Research Institute of Shandong, China) [13].

#### 2.5.3. Determination of Expression Levels of Metabolic Enzymes in CMP

An enzyme-coupling method [37,38] was used to determine the expression levels of NADH in the serum collected at two time points (14:30 and 15:30) and of 12 enzymes in the serum collected at 15:00. The 12 enzymes included hexokinase (HK), glucose-6-phosphate dehydrogenase (G6PDH), triose-phosphate isomerase (TPI), phosphoglycerate kinase (PGK), phosphofructokinase (PFK), aldolase (ALD), glyceraldehyde-3-phosphate dehydrogenase (GAPDH), pyruvate kinase (PK), pyruvate dehydrogenase complex (PDHC), lactate dehydrogenase (LDH), isocitrate dehydrogenase (ICDH), and α-ketoglutarate dehydrogenase complex (α-KGDHC). The detection principles and methods have been described previously [13,39]. The theory was mainly based on the fact that NADH and NADPH absorb light at a wavelength of 340 nm and can generate fluorescence, with excitation and emission wavelengths at 340 nm and 460 nm, respectively. However, NAD+ and NADP+ do not have these characteristics. Thus, we can couple the enzymatic reaction during detection with these two dehydrogenation reactions. A fluorescence-chemiluminescence detector (Fluoroskan Ascent FL, Thermo Fisher Scientific, MA, USA) was used to monitor the kinetic change of fluorescence with a kinetic interval of 15 s and a duration of 3 min. Moreover, with consistent determination conditions and sufficient substrate concentration, enzyme expression levels are proportional to the rate of enzyme catalysis. Therefore, the enzyme expression levels were all measured at 25 °C. Referring to Pierce and Crawford’s [38] method, the enzyme reaction system was used. Total protein in the serum was measured by the Coomassie Brilliant Blue G-250 method. The unit of enzyme expression level is △ mmol NADH or NADPH/ (min·mg protein), expressed as U/g.

### 2.6. Statistical Analysis

Statistical analysis of all data was performed using the SPSS 21.0 (Chicago, IL, USA). The data were analyzed with the Shapiro–Wilk test for normal distribution detection. Then, the comparative *t*-test and the Wilcoxon signed ranks test were used to assess the statistical significance (*p* < 0.05) for parameter data and nonparametric data, respectively. All data were expressed as the mean ± SEM. The correlation analysis of significantly changed cytokines (versus control) and metabolic enzymes was assessed by Pearson’s correlation test.

## 3. Results

All volunteers remained in good health during the experiment. They all strictly complied with the dietary and behavioral requirements of the experiment.

### 3.1. Serum Cytokines Concentrations

**Figure 2:**
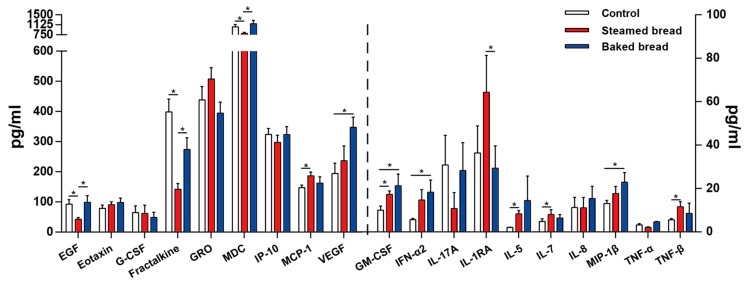
Serum cytokine concentrations. Values are mean ± SEM. * *p* < 0.05.

Concentrations of nineteen cytokines are shown in Figure 2. Nineteen other cytokines were not detected because the concentration of them was below the minimum detectable concentrations. Compared with the control, the intake of steamed bread led to an increase in the concentrations of GM-CSF (the concentration change rate was 48.1%; *p* = 0.001), IL-5 (75.7%; *p* = 0.007), IL-7 (26.2%; *p* = 0.033), MCP-1 (26.3%; *p* = 0.008), and TNF-β (108.0%; *p* = 0.045), and a decrease in the concentrations of EGF (−54.4%; *p* = 0.003), fractalkine (−64.4; *p* = 0.001) and MDC (−23.4%; *p* = 0.001). The intake of baked bread increased the concentrations of GM-CSF (52.6%; *p* = 0.025), IFN-α2 (75.0%; *p* = 0.044), MIP-1β (41.9%; *p* = 0.015), and VEGF (42.6%; *p* = 0.035) as compared with the control. A comparison with the steamed bread reveals that the baked bread increased the concentrations of EGF (*p* = 0.040), fractalkine (*p* = 0.017), and MDC (*p* = 0.026), and decreased the concentration of IL-1RA (*p* = 0.004).

### 3.2. Serum Metabolic Enzyme Expression Levels

**Figure 3:**
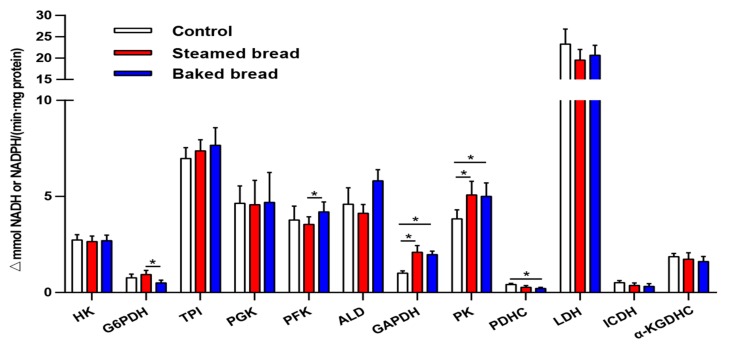
The expression levels of 12 enzymes in the serum. Values are mean ± SEM. * *p* < 0.05.

The expression levels of 12 metabolic enzymes are shown in Figure 3. Compared with the control, the steamed bread increased the expression levels of GAPDH (*p* = 0.042) and PK (*p* = 0.042); the baked bread increased the expression levels of GAPDH (*p* = 0.004) and PK (*p* = 0.037), and decreased the expression levels of PDHC (*p* = 0.006). Compared with the steamed bread, the baked bread decreased the expression level of G6PDH (*p* = 0.033) and increased the expression level of PFK (*p* = 0.029).

### 3.3. Cytokine Network and CMP Metabolic Network

#### 3.3.1. Cytokine Network

**Figure 4:**
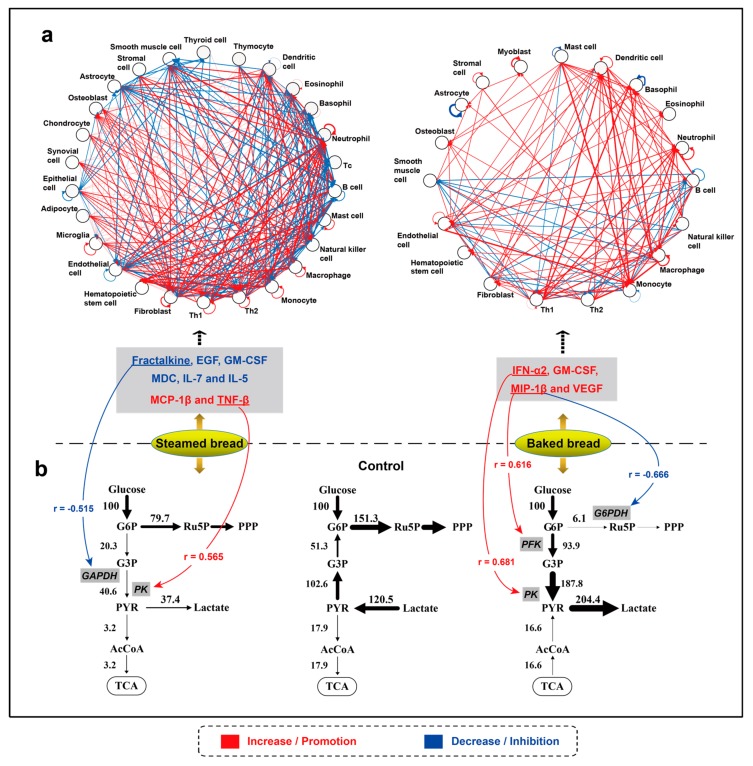
Effects of steamed bread and baked bread on the cytokine network (**a**) and central metabolic pathway metabolic network (**b**). In the cytokine network, the thickness of the lines represents the strength of intercellular communication (the concentration change rate of cytokine *i*); the red line represents enhanced communication, and the blue line represents inhibited communication; the arrow represents the direction from secretory cell to the target cell. In the metabolic network, the thickness of the line represents the quantitative metabolic pathway flux; the arrow represents the direction of metabolic flux. The correlation analysis between the changed (versus control) cytokines and the metabolic enzymes were assessed by Pearson’s correlation test. The correlation is given by *r* value. The red line represents a significant (*p* < 0.05) positive correlation and the blue line represents a significant (*p* < 0.05) negative correlation.

It is hard to grasp the actual functions of single cytokines. An integral understanding of cytokine signaling in the form of a network is essential for greater insight into intercellular communication and systemic immune responses. The network after the intake of steamed bread, including 26 nodes and 331 lines, was red (up-regulation) on the whole (Figure 4a), and the total network strength *St* was 36.39 (Table 2). The network after the intake of baked bread, including 19 nodes and 129 lines, was red on the whole (Figure 4a), and the *St* was 98.54. The input and output strengths of each node (secretory or target cell) are summarized in Table 2. Through the input node strength, the potential degree to which immune and nonimmune cells are activated or inhibited can be obtained. For example, compared with the steamed bread, the intake of baked bread may activate the T helper 1 cell (Th1) (2.60 versus −2.15), monocyte (7.50 versus 4.65), macrophage (8.91 versus 4.65), and natural killer cell (1.26 versus -2.11). In addition to the immune cells, the intake of baked bread may inhibit fibroblast (3.16 versus 11.89) and hematopoietic stem cells (3.16 versus 11.83) as compared with steamed bread.

#### 3.3.2. CMP Metabolic Network

The concentrations of glucose, lactate, and NADH in the serum collected at 14:30 and 15:30 are shown in Table 3. After the matrix calculation, we obtained the CMP metabolic network flux diagram (Figure 4b). After the intake of steamed bread, the lactate metabolic flux increased to 37.4 from −120.5; and the flux entering PPP was reduced to 79.7 from 151.3. After the intake of baked bread, the lactate metabolic flux was 204.4, and the PPP metabolic flux was 6.1. These values indicate that compared with the intake of steamed bread, the intake of baked bread increased the lactate metabolic flux but decreased the PPP metabolic flux.

#### 3.3.3. Relationship Between the Cytokine and CMP Metabolic Network

We analyzed the correlation between significantly changed cytokines (versus control) and metabolic enzymes. The *r* value of the correlation was between −1 and 1. The closer the absolute value of the *r* value was to 1, the stronger the correlation between the two analyzed indexes was. After the ingestion of steamed bread, the concentration of fractalkine was negatively correlated (*r* = −0.515, *p* = 0.041) with the expression level of GAPDH, and the concentration of TNF-β was positively correlated (*r* = 0.565, *p* = 0.022) with the expression level of PK. After the ingestion of baked bread, the concentration of MIP-1β was negatively correlated (*r* = −0.666, *p* = 0.005) with the expression level of G6PDH and positively correlated (*r* = 0.616, *p* = 0.006) with the expression level of PFK, and the concentration of IFN-a2 was positively correlated (*r* = 0.681, *p* = 0.003) with the expression level of PK.

## 4. Discussion

Having a good understanding of the effects of differently processed foods on immunity and metabolism in the human body is important when providing guidance on a healthy diet. The current experiment compared the effects of steamed bread and baked bread made with one piece of fermented wheat dough on the cytokine network and CMP network in the human body. The results showed that compared with steamed bread, baked bread enhanced the intercellular communication/immune response, increased catabolism, and decreased anabolism. These findings highlight the functional differences of foods processed by different methods. Furthermore, the correlation analysis suggests a potential relationship between the intercellular communication network and the metabolic network.

The effects of foods and drugs are reduced with increases in the application duration [24]. This means that repeated and long-term stimuli result in gradual adaptation or tolerance. This notion is supported by the studies on Chinese medicine and on functional foods like resistant starch [24,40]. Therefore, the initial effects of foods should be focused on when evaluating their functions. In this study, volunteers mainly ate rice daily and rarely ate wheat flour foods. Therefore, we tried to evaluate the initial influences of steamed bread and baked bread on the human body. Probably because of the initial effects of these two staple foods, immune response and catabolism were enhanced after the intake of both steamed bread and baked bread (versus control with rice as the staple food), as reflected by the activated immune cells (including monocyte, macrophages, and granulocytes) and the increased EMP metabolic level (including the increased expression levels of PK and GAPDH and the increased lactate metabolic flux), respectively. This is mainly because when a relatively unfamiliar food is ingested, the body goes on alert with a triggered immune response and increased catabolism. However, in the comparison between steamed bread and baked bread, we found different effects of these two staple foods on the human body.

### 4.1. Baked Bread Enhanced the Immune Response as Compared with Steamed Bread

Cytokines are the messengers for regulating immunity, and they are influenced by foods [8]. In this study, steamed bread and baked bread induced different changes in cytokine profiles, suggesting that these two staple foods have different effects on immunity. Compared with steamed bread, the intake of baked bread induced increases in fractalkine and MDC, and a decrease in IL-1RA. Fractalkine/CX3CL1 and MDC/CCL-22; these are all chemokines and can induce directed chemotaxis in immune cells, such as monocytes and natural killer cells [41]. IL-1RA, a physiological antagonist of proinflammatory mediators such as IL-1β, has an important role in immune tolerance [20]. These suggest that baked bread may enhance the immune response as compared with steamed bread.

The cell is the basic unit of biological function, and cytokines execute their functions only through the cells [15]. To explore which cells may be involved in immune responses and understand the interaction between the cells, an intercellular communication network was constructed. Compared with steamed bread, the increased total network strength with the intake of baked bread supported the potential enhanced immune response. Many kinds of innate immune cells, including monocytes, macrophages, and natural killer cells, may be activated with the intake of baked bread, as the input strengths of these cells were higher with baked bread than with steamed bread. Moreover, Th1, the primary regulator of innate and cellular immunity [42], may also be activated with the intake of baked bread as compared with steamed bread. This suggests that baked bread may mainly enhance innate and cellular immune responses as compared with steamed bread.

Different food-processing methods can cause different chemical changes depending on the treatment temperature, humidity, and other factors [1]. When baking at higher temperatures, the Maillard reaction and other chemical reactions can occur. The formation of acrylamide, 5-hydroxymethylfurfural, and other harmful components is the main consequence of the Maillard reaction [43,44]. However, during steaming, with its relatively low temperature and high humidity, the above harmful components may not be produced. Therefore, we speculate that the exposure of potentially harmful compounds to the intestinal mucosa may be the main reason for the enhanced systemic immune response with the intake of baked bread as compared with steamed bread.

In addition to the immune system, cytokines are also involved in regulating the nonimmune functions. The two staple foods potentially affected hematopoiesis, collagen production, and other functions. Interestingly, compared with steamed bread, the intake of baked bread inhibited hematopoietic stem cells and fibroblasts, suggesting that for people who eat wheat flour-based foods, steamed bread may have the potential to promote hematopoiesis and wound healing, as hematopoietic stem cells and fibroblasts are associated with hematopoiesis and wound healing, respectively.

### 4.2. Baked Bread Increased the Catabolism and Decreased the Anabolism as Compared with Steamed Bread

The metabolism of the human body is greatly affected by food [24]. The lactate cycle and blood lactate flux are important indicators of catabolism and oxidative phosphorylation [22,23]. The metabolic flux of PPP reflects anabolic levels since it provides the raw materials and reduces power for anabolic reactions [25]. In comparison with steamed bread, the increase in lactate metabolic flux and the decrease in PPP metabolic flux with the intake of baked bread suggests that catabolism was increased and anabolism was decreased. Metabolic flow is catalyzed by enzymes. PFK is an important rate-limiting enzyme in the EMP pathway [45]. Thus, the increased expression of PFK with the intake of baked bread (versus steamed bread) may be the main reason for the increased EMP metabolic flux. The increased EMP flux may contribute to the increase in lactate metabolic flux. G6PDH is responsible for catalyzing the first-step reaction in PPP. The decreased expression of G6PDH with the intake of baked bread (versus steamed bread) may reduce the metabolic flux entering into PPP. Therefore, this indicates that the difference in CMP metabolic flux after the intake of steamed bread and baked bread may result from the difference in the expressions of the metabolic enzymes PFK and G6PDH. Moreover, these changes in metabolic enzymes further confirmed that catabolism was increased and anabolism was decreased with the intake of baked bread relative to steamed bread.

According to the metabolic findings, for people who mainly live on wheat flour-based foods, baked bread may be helpful for body-weight control, while steamed bread may be more suitable for people suffering from malnutrition or food shortages. Furthermore, compared with baked bread, the increased anabolism with the intake of steamed bread may provide the material support for a potentially enhanced hematopoietic function.

### 4.3. Steamed Bread and Baked Bread May Affect the Secretion of Cytokines Which Regulate Metabolism

The effect of foods on human metabolism comes not only from material and energy (i.e., nutrients), but also information (i.e., non-nutrients) [8,9]. Through the intercellular communication network, the “signal” produced by the exposure of food components to the intestinal mucosa affects the expression of metabolic enzymes [8,24]. On the other hand, the burgeoning field of immunometabolism, which focuses on the interplay between immunological and metabolic processes, indicates that the activation of immunity, especially innate and cellular immunity, must be accompanied by the activation of aerobic glycolysis to provide energy [46,47]. Therefore, there may be a causal relationship between immunity and metabolism; that is to say, cytokines carry the signal to regulate intercellular communication and immune response, and by doing so regulate metabolism. Generally, the energy provided by steamed bread and baked bread are almost all absorbed in the form of glucose, which, in itself, could hardly cause the differences in metabolism. Therefore, the different chemical compositions produced by steaming and baking may regulate metabolism by affecting intercellular communication and immune responses. After the correlation analysis, we inferred that with the intake of steamed bread, the increase in TNF-β may increase the expression of PK, and the decrease in fractalkine may increase the expression of GAPDH. With the intake of baked bread, the increase in MIP-1β may increase the expression of PFK and decrease the expression of G6PDH, and the increase in IFN-α2 may increase the expression of PK. This suggests that steamed bread and baked bread may affect the secretion of cytokines, resulting in the regulation of metabolism in the human body.

In addition, the intermediate signaling pathways that the foods affect are worthy of further study, especially the effects of foods on intestinal mucosa. This will have an important implication for uncovering the mechanism by which food functions. Phosphoproteomics may be of some help, and at present, we are conducting relevant work in this regard.

Collectively, in this study, the intake of baked bread enhanced immune response and increased catabolism as compared with steamed bread. Further, from the perspective of food-processing methods, compared to foods processed by steaming, foods processed by baking may tend to enhance immune response and increase catabolism in the human body, but more work is needed to support this conclusion.

Of course, this study also has certain limitations. Given the specific recipes used in this study, it may not be appropriate to extrapolate our results to other recipes. Moreover, subjects were all young and healthy volunteers with specific eating patterns; thus, it may not be possible to extrapolate our findings to all people, especially those with diseases, different eating habits, or those of other ages. Therefore, more extensive research is needed in the future. Although the evaluation of the short-term or initial effects of the foods is essential, the short-term effects do not reflect the longer-term effects or clinical outcomes. Thus, the long-term effects of these two staple foods on immune and nonimmune functions also need further observation. However, the findings in this study provided critical evidence for understanding the effects of foods processed by different methods on the physiological functions of the human body. In addition, the two network models used in this study simplified complex immune- and metabolic-related biological processes. These two models need to be continuously improved, including the cytokine database and the metabolic pathway (e.g., through the addition of the ketone body metabolic pathway). Nevertheless, the establishment of these two models may provide a systematic and quantitative evaluation method for food functions.

## 5. Conclusions

Baked bread and steamed bread have different impacts on the cytokine network and CMP network in the human body. Compared with steamed bread, the intake of baked bread enhanced intercellular communication and immune response, increased catabolism, and decreased anabolism. Cytokines may act as signals that regulate metabolism. This study provides novel insights into whether processing methods affect the physiological functions of foods and feasible methods regarding how these functions can be evaluated in the human body.

## Figures and Tables

**Table 1:** Metabolic flux model equation.

Intermediate Metabolites	Mass Balance Equations
Glucose-6-phosphate	X_1_ = r1 − r2 − r3
Glyceraldehyde-3-phosphate	X_2_ = r3 − r4
Pyruvate	X_3_ = r4 − r5 − r6
Acetyl Coenzyme A	X_4_ = r6 − r7

**Table 2:** The input and output strengths of each cell and the total strength in the cytokine network. ^1.^

	Immune Cells	Output/Input		Nonimmune Cells	Output/Input		Total Network Strength (*St*)
		Nodes Strength ^2^	Nodes Number		Nodes Strength ^2^	Nodes Number	
**Steamed Bread**	Th1	2.18/−2.15	15/23	Fibroblast	−3.99/11.89	21/20	36.39
	Th2	3.72/−6.65	15/22	Hematopoietic stem cell	0/11.83	0/15	
	Monocyte	0.24/4.65	19/22	Endothelial cell	−1.27/−7.20	18/15	
	Macrophage	0.24/4.65	19/22	Smooth muscle cell	0.52/−5.15	19/8	
	Natural killer cell	−3.90/−2.11	16/19	Osteoblast	2.63/4.38	10/14	
	B cell	0.24/−3.34	19/17	Astrocyte	−5.12/−2.18	16/4	
	Neutrophil	−3.24/15.87	9/15	Stromal cell	2.70/0	14/0	
	Eosinophil	−0.12/4.27	12/23	Thyroid cell	0/−2.18	0/4	
	Basophil	4.14/−6.86	14/18	Epithelial cell	−0.09/−2.18	15/4	
	Dendritic cell	0.33/10.10	18/13	Synovial cell	2.63/0	10/0	
	Mast cell	5.36/−0.80	14/22	Chondrocyte	2.63/0	10/0	
	Cytotoxic T lymphocyte	0/−11.21	0/13	Adipocyte	2.63/0	10/0	
	Microglia	2.63/2.56	10/18				
	Thymocyte	3.09/0	12/0				
**Baked Bread**	Th1	6.31/2.60	14/10	Fibroblast	0/3.16	10/6	98.54
	Th2	6.31/−4.5	12/6	Hematopoietic stem cell	0/3.16	0/6	
	Monocyte	2.10/7.50	11/12	Endothelial cell	6.31/−0.07	12/12	
	Macrophage	10.11/8.91	17/10	Smooth muscle cell	1.70/−4.5	4/6	
	Natural killer cell	0/1.26	10/3	Osteoblast	0/3.16	0/6	
	B-cell	2.10/−1.34	11/10	Astrocyte	0/1.28	0/3	
	Neutrophil	0/8.91	0/10	Stromal cell	1.70/0	4/0	
	Eosinophil	0/3.16	0/6	Myoblast	0/1.28	0/3	
	Basophil	0/7.66	0/10				
	Mast cell	6.31/0	12/0				
	Dendritic cell	6.31/7.66	12/10				

^1^ Original computing matrices are shown in the Supplementary Materials. ^2^ Node strength was the total of the strength of the adjacent edges.

**Table 3:** Serum glucose, lactate, and NADH levels.

Items		Control	Steamed Bread	Baked Bread
**Serum collected at 14:30**	Glucose (mg/dL)	89.14 ± 2.99	88.94 ± 2.46	87.67 ± 2.88
	Lactate (mg/dL)	22.76 ± 0.88	21.00 ± 0.85	24.63 ± 1.01
	NADH (mg/L)	38.70 ± 7.29	69.26 ± 12.54	33.31 ± 6.23
**Serum collected at 15:30**	Glucose (mg/dL)	91.78 ± 1.65	79.15 ± 1.61	84.29 ± 2.76
	Lactate (mg/dL)	19.57 ± 0.91	17.35 ± 0.82	17.73 ± 0.95
	NADH (mg/L)	62.29 ± 9.40	53.73 ± 9.65	61.51 ± 8.55

## Supplementary Materials

The following are available online at https://www.mdpi.com/2072-6643/12/1/1/s1, Table S1: The computing matrices of cytokine network.

## Abbreviations

α-ketoglutarate dehydrogenase complex; ALD, aldolase; EGF, epidermal growth factor; EMP, Embden-Meyerhof-Parnas pathway; G3P, glyceraldehyde-3-phosphate; G6PDH, glucose-6-phosphate dehydrogenase; GAPDH, glyceraldehyde-3-phosphate dehydrogenase; G-CSF, granulocyte colony-stimulating factor; GM-CSF, granulocyte macrophage-colony-stimulating factor; GRO, growth-related oncogene; HK, hexokinase; ICDH, isocitrate dehydrogenase; IFN, interferon; IL, interleukin; IP-10, interferon-inducible protein-10; LDH, lactate dehydrogenase; MCP, monocyte chemotactic protein; MDC, macrophage-derived chemokine; MIP, macrophage inflammatory protein; PDHC, pyruvate dehydrogenase complex; PFK, phosphofructokinase; PGK, phosphoglyceric kinase; PK, pyruvate kinase; PPP, pentose phosphate pathway; TCA cycle, tricarboxylic acid cycle; Th, T helper cell; TNF, tumor necrosis factor; TPI, triose-phosphate isomerase; VEGF, vascular endothelial cell growth factor.
